# Clinical Symptoms, Neurological Signs, and Electrophysiological Findings in Surviving Residents with Probable Arsenic Exposure in Toroku, Japan

**DOI:** 10.1007/s00244-018-0544-8

**Published:** 2018-07-04

**Authors:** Nobuyuki Ishii, Hitoshi Mochizuki, Yuka Ebihara, Kazutaka Shiomi, Masamitsu Nakazato

**Affiliations:** Division of Neurology, Respirology, Endocrinology and Metabolism, Department of Internal Medicine, University of Miyazaki, 5200 Kihara, Kiyotake, Miyazaki, 889-1692 Japan

## Abstract

Chronic arsenic intoxication is known to cause multisystem impairment and is still a major threat to public health in many countries. In Toroku, a small village in Japan, arsenic mines operated from 1920 to 1962, and residents suffered serious sequelae of arsenic intoxication. We have performed annual medical examinations of these residents since 1974, allowing us to characterize participants’ long-term health following their last exposure to arsenic. The participants could not be described as having “chronic arsenic intoxication,” because their blood arsenic levels were not measured. In this study, we defined them as having “probable arsenic intoxication.” Symptoms frequently involved the sensory nervous system, skin, and upper respiratory system (89.1–97.8%). In an analysis of neurological findings, sensory neuropathy was common, and more than half of the participants complained of hearing impairment. Longitudinal assessment with neurological examinations and nerve conduction studies revealed that sensory dysfunction gradually worsened, even after exposure cessation. However, we could not conclude that arsenic caused the long-term decline of sensory function due to a lack of comparisons with age-matched healthy controls. This is the first study to characterize the longitudinal sequelae after probable arsenic exposure. Our study will be helpful to assess the prognosis of patients worldwide who still suffer from chronic arsenic intoxication.

Arsenic is a heavy metal that causes various adverse effects on human health. It accumulates in the body and generates reactive oxygen species, resulting in oxidative damage to vital organs (Abdul et al. 2015). Chronic arsenic accumulation eventually leads to psychological effects, decreased mental performance, peripheral neuropathy, hypertension, and increased risks of cardiovascular disorders, diabetes, and cancer (Guha Mazumder and Dasgupta 2011; Rehman et al. 2018). However, no previous studies have described the long-term sequelae of arsenic intoxication after exposure cessation.

Arsenic intoxication, especially when chronic, is still a major threat to public health; at least 140 million people in 50 countries are exposed to contaminated drinking water (World Health Organization 2017). Chronic arsenic toxicity, which is due to low-concentration exposure over a long period of time, impairs the same organs and tissues as acute toxicity, although in cases of higher exposure levels, skin and nervous system disturbances are usually more pronounced (Lagerkvist and Zetterlund 1994; Ahsan et al. 2006; Naujokas et al. 2013). Chronic arsenic intoxication has been caused not only by drinking contaminated groundwater (Mazumder et al. 1998; Milton and Rahman 2002; Chakraborti et al. 2003; Otto et al. 2006; Chen 2014; Del Rio et al. 2017; Rehman et al. 2018) but also by occupational exposures, such as mining (Ishinishi et al. 1977; Kawasaki et al. 2002; Eisler 2004) and work with copper smelters (Feldman et al. 1979; Lubin et al. 2008; Halatek et al. 2009; Sinczuk-Walczak et al. 2010).

In Toroku, a small village in a narrow valley in Miyazaki prefecture, Japan, where approximately 300 residents originally lived, arsenic mines were in operation from 1920 to 1962, except for several years around 1945 because of World War II. Arsenic was roasted at a refinery without a dust-collecting system. Thus, huge amounts of arsenic trioxide-containing gases were leaked from the roasters and intermittently covered the entire Toroku valley, causing both refinery workers and residents to be exposed to arsenic orally (via contaminated food and water), dermally, or tracheally (via contaminated air) (Miyazaki prefecture 1972; Tsuda et al. 1990). The polluted environment caused chronic arsenic intoxication in many workers and residents, leading to many deaths before 1962 (Miyazaki prefecture 1972; Mochizuki et al. 2016). In 1972, 10 years after the mine closed, arsenic concentrations were finally measured by Miyazaki prefecture. Extremely high arsenic content (200–8000 mg/kg) was detected in the dust of ceiling boards of residences near the mine, and the average arsenic concentrations in neighboring soil and in water percolating from the slag were 2760 mg/kg and 180 mg/L, respectively. In the residents of Toroku, high arsenic concentrations were also detected in the hair (average concentration, 1.52 mg/kg; n = 29), fingernails (10.83 mg/kg; n = 31), and urine (0.58 mg/L; n = 38) (Miyazaki prefecture 1972; Hotta et al. 1979), presumably because many residents had continued to drink the contaminated water from Toroku River after air exposure had ceased. Beginning in 1974, surviving residents have undergone the annual Toroku Medical Examination, which has provided us with more than 40 years of longitudinal information about the clinical characteristics of arsenic intoxication. However, it is difficult to define the participants as having “chronic arsenic intoxication,” because the arsenic concentrations in their hair, fingernails, and urine were measured only once in a portion of residents at 10 years after exposure cessation, and their blood arsenic levels have not been measured. In this paper, we defined the residents in Toroku as having “probable arsenic intoxication.”

To date, there have been no reports describing the long-term medical history of patients who are no longer exposed to arsenic; most previous studies have been cross-sectional, and patients were still actively exposed to arsenic (Rehman et al. 2018). Additionally, the health effects of arsenic exposure during infancy and early childhood remain unclear. To clarify the long-term medical history of patients with arsenic intoxication after exposure cessation, we conducted the following investigations in Toroku residents with probable arsenic intoxication: (1) a descriptive analysis of symptoms, comorbidities, and neurological findings; (2) an observational analysis of sex differences in participants with probable arsenic intoxication during both adulthood and during infancy and early childhood; and (3) a longitudinal analysis of sensory dysfunction based on physical and neurophysiological examinations.

## Materials and Methods

### Descriptive Study

All participants enrolled in this study lived within 1000 m of the mine roaster prior to 1962, had typical symptoms and signs of arsenic poisoning, such as dermatological manifestations (pigmentation or keratosis) or neurological disturbances (peripheral neuropathy), and were diagnosed with chronic arsenic exposure by the government after 1974 when administration of the Toroku Medical Examination began. Because no participants in this study underwent measurement of blood arsenic levels, we defined them as having probable arsenic intoxication.

### Questionnaire

From 1974 to 2005, all participants were administered a self-reported questionnaire asking whether they had experienced certain subjective symptoms or comorbidities that lasted for more than 1 year. The subjective symptoms comprised sensory disturbance, skin disturbance, upper and lower respiratory symptoms, hearing symptoms, visual symptoms, headache, dizziness, gastrointestinal symptoms, vascular disturbance, teeth problems, olfactory dysfunction, tremor, anemia, liver/gallbladder symptoms, wasting, and taste disturbance. The comorbidities comprised neuropathy/radiculopathy, hypertension, respiratory disorders, gastrointestinal disorders, cardiac disease, cataracts, skin disease, liver disease, and diabetes mellitus. We analyzed the relationship between these parameters, sex, and arsenic exposure during infancy and early childhood. We defined “infancy and early childhood” as the period from age 0 to 9 years and evaluated the long-term effect of arsenic exposure in participants who spent at least 1 year of their infancy and/or early childhood in Toroku.

### Evaluation of Sensory Dysfunction

We evaluated sensory impairment with a sharp toothpick for pain, test tubes containing water at 0 or 50 °C for temperature, soft tissue paper for touch, and a 128 Hz tuning fork for vibration. The severity of sensory disturbances was graded as follows: “0,” almost normal sensation; “1,” sensation decreased by less than half; and “2,” sensation decreased by more than half.

### Longitudinal Study of Sensory Dysfunction

To evaluate long-term sensory dysfunction by neurological examination, we selected participants who had undergone the Toroku Medical Examination more than ten times for more than 10 years. For nerve conduction studies (NCS), which were first conducted in 2003, we enrolled participants if they had undergone NCS more than three times. For both types of analyses, participants were excluded if they had diabetes mellitus, alcoholism, or a past history of gastric resection.

### NCS Protocol

NCS were conducted using a Neuropack MEB2200 electromyogram apparatus (Nihon Kohden, Tokyo, Japan). Motor NCS were performed on the median and tibial nerves. The stimulus site was 3 cm proximal to the wrist crease and elbow in median nerve studies and just lateral to the medial malleolus and popliteal fossa in tibial nerve studies. Antidromic sensory NCS were conducted on the median and sural nerves. In the median nerve study, the recording site was the index finger with the stimulation at the wrist, and in the sural nerve study, the recording site was the lateral malleolus with stimulation 14 cm proximal to the mid-calf.

This study protocol was approved by the Ethics Committee of the University of Miyazaki, with a waiver of written, informed consent obtained from participants with chronic arsenic exposure, and was performed in accordance with the Declaration of Helsinki.

### Statistical Analysis

Categorical variables are shown as percentages (%), whereas continuous variables are shown as medians (25th–75th percentiles). The Fisher’s exact test and the Wilcoxon rank-sum test were used for between-group comparisons of categorical and continuous data, respectively. A linear mixed-effect model analysis for repeated measurements within participants was used for continuous outcome measures (severity of superficial and deep sensations, and amplitude and velocity in the NCS). The fixed effects were the numbers of years the Toroku Medical Examination was administered, whereas the random effect was the participant. The statistical significance level was set at *P* = 0.05. Statistical analyses were performed using EZR (version 1.36, Saitama Medical Center, Jichi Medical University, Saitama, Japan) (Kanda 2013), which is a graphical user interface for R software (version 3.4.1, R Development Core Team; https://www.r-project.org/).

## Results

### Participant Characteristics

The participant characteristics are summarized in Table 1. We enrolled 137 consecutive participants with probable arsenic intoxication; 70 (51.1%) were male, and 91 (66.4%) had an occupational history that included work at the arsenic mine. Eighty-nine participants (65.4%) were exposed to arsenic during infancy and early childhood, as defined in the Methods. Workers at the arsenic mine were more likely to be male than female (*P* = 0.01).

**Table 1:** Participant characteristics

Parameters	Total (*n* = 137)	Male (*n* = 70)	Female (*n* = 67)	*P* value
Previously worked at the arsenic mine, *n* (%)	91 (66.4)	54 (77.1)	37 (55.2)	0.01*
Initial age of arsenic exposure, year	0 (0–16)	0 (0–48)	1 (0–42)	0.85
Participants with arsenic exposure during infancy and early childhood, *n* (%)	89 (65.4)	51 (72.9)	38 (57.6)	0.07
Participants exposed before 1942, *n* (%)	107 (83.6)	58 (87.9)	49 (79.0)	0.23
Age in 1962 when the mine closed	42 (34.0–51.0)	43 (16–62)	41 (20–61)	0.87
Survivors in 2014, *n* (%)	27 (19.7)	15 (21.4)	12 (17.9)	0.67
average age, year (survivors, *n* = 27)	81 (77.5–83.5)	81.8 (67.6–93.9)	81.4 (72.1–91.8)	0.88
average age of death, year (non-survivors, *n* = 110)	79 (70.3–85.0)	75.1 (55.6–92.9)	80.8 (56.7–97.8)	0.05

*P < 0.05, categorical variables are shown as numbers (percentages) and continuous variables are shown as medians (25th–75th percentiles)

### Questionnaire Results: Subjective Symptoms and Comorbidities

Table 2 summarizes the cumulative questionnaire data regarding subjective symptoms and comorbidities. Sensory disturbance was the most common symptom (97.8%), followed by skin disturbance (95.6%), upper respiratory symptoms (90.5%), hearing (89.1%), and visual symptoms (89.1%). Frequent comorbidities included neuropathy/radiculopathy (43.1%), hypertension (33.6), respiratory disease (32.1%), and gastrointestinal disorders (29.2%). Twenty participants (14.6%) had no comorbidities. In an analysis of the questionnaire data by sex, the only significant finding was that cataracts were significantly more prevalent in females (*P* = 0.04).

**Table 2:** Subjective symptoms and comorbidities from questionnaire: cumulative data

Parameters	Total (*n* = 137)	Male (*n* = 70)	Female (*n* = 67)	*P* value
Subjective symptoms				
Sensory disturbance	134 (97.8)	68 (97.1)	66 (98.5)	1.00
Skin disturbance	131 (95.6)	67 (95.7)	64 (95.5)	1.00
Upper respiratory symptoms	124 (90.5)	62 (88.6)	62 (92.5)	0.56
Hearing symptoms	122 (89.1)	64 (91.4)	58 (86.6)	0.42
Visual symptoms	122 (89.1)	60 (85.7)	62 (92.5)	0.28
Headache	120 (87.6)	58 (82.9)	62 (92.5)	0.12
Dizziness	117 (85.4)	60 (85.7)	57 (85.1)	1.00
Gastrointestinal symptoms	114 (83.2)	57 (81.4)	57 (85.1)	0.65
Lower respiratory symptoms	113 (82.5)	60 (85.7)	53 (79.1)	0.37
Vascular disturbance	105 (76.6)	52 (74.3)	53 (79.1)	0.55
Teeth problems	102 (74.5)	48 (68.6)	54 (80.6)	0.12
Olfactory dysfunction	96 (70.1)	45 (64.3)	51 (76.1)	0.14
Tremor	70 (51.1)	36 (51.4)	34 (50.7)	1.00
Anemia	61 (44.5)	26 (37.1)	35 (52.2)	0.09
Liver/gallbladder symptoms	57 (41.6)	33 (47.1)	24 (35.8)	0.23
Wasting	55 (40.1)	26 (37.1)	29 (43.3)	0.49
Taste disturbance	47 (34.3)	22 (31.4)	25 (37.3)	0.48
Comorbidities				
Neuropathy/radiculopathy	59 (43.1)	25 (35.7)	34 (50.7)	0.09
Hypertension	46 (33.6)	24 (34.3)	22 (32.8)	1.00
Respiratory disorders	44 (32.1)	23 (32.9)	21 (31.3)	0.86
GI disorders	40 (29.2)	22 (31.4)	18 (26.9)	0.58
Cardiac disease	38 (27.7)	24 (34.3)	22 (32.8)	1.00
Cataracts	30 (21.9)	10 (14.3)	20 (29.9)	0.04*
Skin disease	15 (10.9)	9 (12.9)	6 (9.0)	0.59
Liver disease	15 (10.9)	8 (11.4)	7 (10.4)	1.00
Diabetes mellitus	12 (8.8)	7 (10.0)	5 (7.5)	0.77
No comorbidities	20 (14.6)	8 (11.4)	12 (17.9)	0.34

*P < 0.05, categorical variables are shown as numbers (percentages)

### Objective Neurological Findings

Cumulative data of objective neurological findings are summarized in Table 3. Abnormalities in cranial nerves I (olfactory nerve, 27.7%) and VIII (vestibulocochlear nerve, 50.4%), and sensory dysfunction in the bilateral lower distal limbs (28.5%), were common neurological findings in participants with arsenic intoxication, while 37 participants (27.0%) had no neurological abnormalities. Female participants had a higher prevalence of gait disturbance due to joint problems than male participants (*P* = 0.01).

**Table 3:** Objective neurological findings: cumulative data

Parameters	Total (*n* = 137)	Male (*n* = 70)	Female (*n* = 67)	*P* value
Memory impairment	22 (16.1)	11 (15.7)	11 (16.4)	1.00
Frontal release sign	4 (2.9)	0 (0.0)	4 (6.0)	0.06
Cranial nerves				
I	38 (27.7)	22 (31.4)	16 (23.9)	0.35
II	12 (8.8)	4 (5.7)	8 (11.9)	0.24
III	1 (0.7)	0 (0.0)	1 (1.5)	0.45
IV	0 (0.0)	0	0	NA
V	0 (0.0)	0	0	NA
VI	0 (0.0)	0	0	NA
VII	5 (3.6)	3 (4.3)	2 (3.0)	1.00
VIII	69 (50.4)	32 (45.7)	37 (55.2)	0.31
IX	0 (0.0)	0	0	NA
X	0 (0.0)	0	0	NA
XI	0 (0.0)	0	0	NA
XII	0 (0.0)	0	0	NA
Motor dysfunction				
Hemiparesis	14 (10.2)	7 (10.0)	7 (10.4)	1.00
Neuropathy	3 (2.2)	3 (4.3)	0 (0.0)	0.23
Tremor	1 (0.7)	1 (1.4)	0 (0.0)	1.00
Parkinsonism	1 (0.7)	0 (0.0)	1 (1.5)	0.49
Sensory dysfunction				
Bilateral distal limbs	39 (28.5)	20 (28.6)	19 (28.4)	1.00
Laterality (hemi)	11 (8.0)	3 (4.3)	8 (11.9)	0.12
Headache	2 (1.5)	1 (1.4)	1 (1.5)	1.00
Cerebellar dysfunction	9 (6.6)	5 (7.1)	4 (6.0)	1.00
Gait disturbance due to				
Sensory abnormalities	14 (10.2)	10 (14.3)	4 (6.0)	0.16
Hemiparesis (stroke)	8 (5.8)	3 (4.3)	5 (7.5)	0.49
Orthopedic problems	6 (4.4)	0 (0.0)	6 (9.0)	0.01*
Parkinsonism	2 (1.5)	0 (0.0)	2 (3.0)	0.24
No neurological abnormalities	37 (27.0)	20 (28.6)	17 (25.4)	0.70

*NA* not analyzed

*P < 0.05, categorical variables are shown as numbers (percentages)

### Clinical Symptoms and Neurological Signs in Participants with Probable Arsenic Exposure During Infancy and Early Childhood

Eighty-nine participants, including 51 males (57.3%) and 38 females (42.7%), were exposed to arsenic during infancy and early childhood, as defined in the *Methods*. Basic characteristics, such as occupational history, age, and the number of survivors in 2014, were not significantly different between male and female participants (data not shown). In the questionnaire (Table 4), female participants had a higher prevalence of olfactory dysfunction than male participants (*P* = 0.04). There were no significant differences in other subjective symptoms (Table 4), comorbidities, or neurological findings (*P* > 0.05; data not shown).

**Table 4:** Subjective symptoms in participants with arsenic exposure during infancy and childhood: cumulative data

Parameters	Total (*n* = 89)	Male (*n* = 51)	Female (*n* = 38)	*P* value
Sensory disturbance	87 (97.8)	49 (96.1)	38 (100.0)	0.51
Skin disturbance	83 (93.3)	48 (94.1)	35 (92.1)	1.00
Upper respiratory symptoms	78 (87.6)	45 (88.2)	33 (86.8)	1.00
Hearing symptoms	78 (87.6)	46 (90.2)	32 (84.2)	0.52
Visual symptoms	78 (87.6)	44 (86.3)	34 (89.5)	0.75
Headache	73 (82.0)	40 (78.4)	33 (86.8)	0.41
Dizziness	75 (84.3)	43 (84.3)	32 (84.2)	1.00
Gastrointestinal symptoms	73 (82.0)	40 (78.4)	33 (86.8)	0.41
Lower respiratory symptoms	72 (80.9)	43 (84.3)	29 (76.3)	0.42
Vascular disturbance	66 (74.2)	38 (74.5)	28 (73.7)	1.00
Teeth problems	65 (73.0)	36 (70.6)	29 (76.3)	0.63
Olfactory dysfunction	62 (69.7)	31 (60.8)	31 (81.6)	0.04*
Tremor	39 (43.8)	23 (45.1)	16 (42.1)	0.83
Anemia	40 (44.9)	20 (39.2)	20 (52.6)	0.28
Liver/gallbladder symptoms	36 (40.4)	23 (45.1)	13 (34.2)	0.38
Wasting	32 (36.0)	18 (35.3)	14 (36.8)	1.00
Taste disturbance	30 (33.7)	15 (29.4)	15 (39.5)	0.37

*P < 0.05, categorical variables are shown as numbers (percentages)

### Objective Sensory Dysfunction and NCS

Of the 128 participants enrolled in the study, the following participants were excluded in the evaluation of longitudinal sensory dysfunction: 12 with diabetes, 3 with a history of gastric resection. No participants were excluded due to alcoholism. The numbers of participants who met the inclusion criteria described in the *Methods* were 28 and 18 for the long-term follow-up of superficial and deep sensation, respectively. The participant characteristics are summarized in Table 5. The durations of follow-up were 30 and 31 years for superficial and deep sensation, respectively. Both superficial and deep sensation worsened significantly during follow-up (*P* = 0.04 and *P* < 0.01, respectively).

**Table 5:** Participant characteristics and a mixed effect model: long-term follow-up of objective sensory dysfunction

Parameters	Objective sensory dysfunction	
	Superficial sensation (*n* = 28)	Deep sensation (*n* = 18)
Men, *n* (%)	11 (39.2)	6 (33.3)
Duration of arsenic exposure, year	38 (32.0–42.0)	29.5 (28.0–35.5)
Age in 1975, year	51 (44.8–55.0)	49 (44.3–53.0)
Survivors in 2014, *n* (%)	14 (50.0)	8 (44.4)
Average age, year (survivors, *n* = 14)	84 (82.2–91.2)	82.5 (80.3–85.3)
Average age of death, year (nonsurvivors, *n* = 14)	86 (77.8–88.0)	86.5 (76.3–88.0)
Follow-up duration, year	29 (27.8–34.0)	36 (31.3–40.0)
Mixed effect model		
Slope	6.16 × 10^−5^*	2.06 × 10^−4^**

*P < 0.05; **P < 0.01, categorical variables are shown as numbers (percentages) and continuous variables are shown as medians (25th–75th percentiles)

**Fig. 1:**
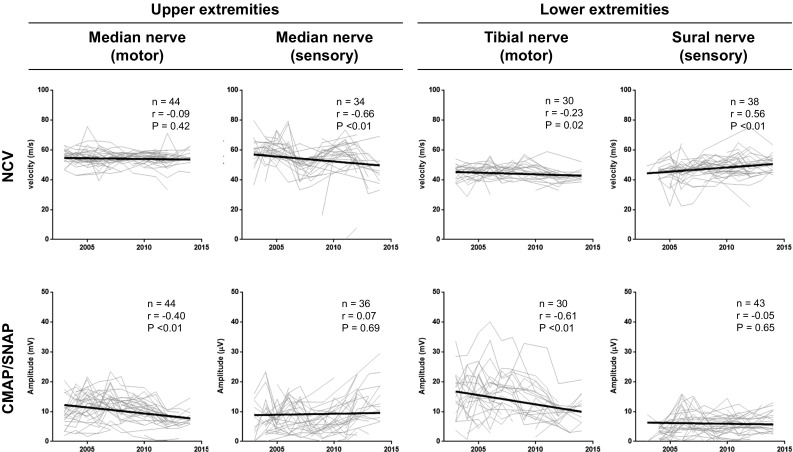
Nerve conduction studies of the upper and lower limbs. In the upper extremities, the compound muscle action potential (CMAP) of the median motor nerve and the nerve conduction velocity (NCV) of the median sensory nerve were significantly worsened. In the lower extremities, the CMAP of the tibial motor nerve deteriorated while the NCV of the sural sensory nerve was significantly ameliorated. *NCV* nerve conduction velocity, *CMAP* compound muscle action potential, *SNAP* sensory nerve action potential

For the NCS, between 30 and 44 participants were enrolled in each test according to the inclusion criteria. Results of the 10-year follow-up are shown in Fig. 1. In the upper extremities, the compound muscle action potential (cMAP) of motor nerves and the nerve conduction velocity (NCV) of sensory nerves worsened significantly during the follow-up period (*P* < 0.01 and *P* < 0.01, respectively). In the lower extremities, the cMAP and NCV of the motor nerves worsened significantly (*P* < 0.01 and *P* = 0.02, respectively). The NCV of the sensory nerves in the lower extremities improved significantly during follow-up (*P* < 0.01).

## Discussion

This study showed that participants who had experienced probable arsenic exposure exhibited various multisystem abnormalities at very high rates even more than 10 years after exposure cessation. The questionnaire revealed that symptoms involving the sensory nervous system, skin, and upper respiratory system were common (89.1–97.8%) and that comorbidities, such as neuropathy/radiculopathy, hypertension, and respiratory disease, also were frequent (32.1–43.1%). In an analysis of neurological findings, sensory neuropathy was common, and more than half of participants complained of hearing impairment. Longitudinal assessment by neurological examination and NCS revealed that sensory dysfunction gradually worsened even after exposure cessation. However, we could not conclude that arsenic caused the long-term decline of sensory function due to a lack of comparisons with age-matched healthy controls.

### Subjective Symptoms, Comorbidities, and Neurological Findings

Participants with probable arsenic exposure demonstrated various symptoms of intoxication even more than 10 years after exposure cessation. Typical symptoms of chronic arsenic intoxication include neurological and dermatological abnormalities; in contrast to acute poisoning, gastrointestinal symptoms were found to be uncommon in a recent review (Naujokas et al. 2013). In our study, almost all participants developed neurological and dermatological symptoms, and 80% had gastrointestinal symptoms. Moreover, about half of all participants had the following uncommon subjective symptoms and neurological abnormalities: dysfunction of the ears, eyes, or mouth; olfactory disturbance; or memory impairment. Differences in exposure routes may impact which symptoms develop. In our participants, contaminated drinking water was less of a problem than direct exposure of the ears, eyes, mouth, or nose to air containing high concentrations of arsenic. Furthermore, previous studies revealed that chronic arsenic exposure caused eye diseases (See et al. 2007; Sinczuk-Walczak et al. 2010; Chen 2014), hearing impairment (Kesici et al. 2016; Li et al. 2017), oral cavity lesions (Sarwar et al. 2010; Syed et al. 2013), olfactory dysfunction (Ishinishi et al. 1977; Mukherjee et al. 2014), and memory impairment (Tyler and Allan 2014). Arsenic levels in some of our participants’ hair were found to be high when the Toroku Medical Examination was first administered (10 years after exposure ended). It is of interest that participants with probable exposure had high rates of various intoxication symptoms even more than 10 years after exposure cessation.

Workers at the arsenic mine were significantly more likely to be male than female. However, there were no differences in symptoms and neurological findings in males and females except for a higher prevalence of both cataracts and gait disturbance due to orthopedic problems in females. This may be because arsenic pollution existed not only in the mines but also throughout the Toroku valley, in both the air and drinking water. It is unknown why female participants in this study were more likely to develop cataracts, because the prevalence of this condition is generally the same for both sexes (Hirvela et al. 1995). Female participants also tended to have orthopedic problems that led to difficulty walking. This may not be related to arsenic intoxication, because women generally have a higher prevalence of osteoarthritis of the knee and hip (O’Connor 2007). In addition, female participants who experienced probable arsenic exposure during infancy and early childhood were more likely to develop olfactory dysfunction. Previous studies of olfactory problems (Ishinishi et al. 1977; Mukherjee et al. 2014) did not compare males and females, and the reason for the sex difference in this study was unclear.

### Longitudinal Follow-Up of Sensory Function

Neurological examination and NCS showed that sensory dysfunction in participants with probable arsenic intoxication worsened over the 40-year follow-up, a period that ended long after the last exposure, although the nerve conduction velocity of the sural nerve gradually improved. With increased age, even people with no comorbidities demonstrate decreased vibration sense in the toes and ankle joints after age 70 (Potvin et al. 1980), as well as reduced superficial sensation (Guergova and Dufour 2011) and impaired NCS (Rivner et al. 2001). In the NCS of participants with chronic arsenic intoxication, Supapong et al. (2004) found no significant difference compared with healthy controls, while other studies demonstrated significantly worse peripheral neuropathy in those exposed to arsenic (Feldman et al. 1979; Lagerkvist and Zetterlund 1994; Mukherjee et al. 2003). Therefore, the impaired sensory function observed in our study may have been age-related rather than due to arsenic intoxication, because the participants were not compared with age-matched controls.

Two different methodologies revealed different results regarding lower limb sensation: NCS of the sural nerve improved, but sensory function evaluated by neurological examination worsened. This discrepancy could not be explained solely by the age-related impairment of large peripheral nerves. Indeed, two studies that evaluated sensory disturbance in residents of Toroku demonstrated central nervous system dysfunction (Mochizuki et al. 2016) and small fiber neuropathy (Kawasaki et al. 2002). Although the detailed mechanisms are unclear, these conditions may have contributed to the gradual sensory impairment.

### Study Limitations

There were several limitations to this study. First, the participants’ blood arsenic levels were not measured, and therefore arsenic intoxication was probable rather than definite. Second, almost all participants were elderly, and we did not compare them with age-matched controls. Therefore, results of the longitudinal analysis could not exclude the effect of aging. Third, a considerable number of patients with severe arsenic intoxication were not enrolled because some died before 1962. Thus, some results in this study were less severe than they might have been if patients with more serious conditions had been included. Finally, the exact duration of the arsenic exposure could not be identified because only minimal information was recorded during World War II (1939–1945). Thus we could not analyze the relationship between exact exposure levels and durations on the one hand, and symptoms and neurological findings on the other.

## Conclusions

Participants with probable arsenic exposure had high rates of various multisystem abnormalities, even 40 years after exposure cessation. This is the first study to demonstrate the longitudinal sequelae of probable arsenic intoxication long after exposure cessation. Our study will be helpful for assessing the prognosis of patients worldwide who still suffer from chronic arsenic intoxication.
